# MiR-221, a potential prognostic biomarker for recurrence in papillary thyroid cancer

**DOI:** 10.1186/s12957-016-1086-z

**Published:** 2017-01-07

**Authors:** Lei Dai, Yaozong Wang, Liangliang Chen, Jueru Zheng, Jianjun Li, Xianjiang Wu

**Affiliations:** Department of Thyroid Surgery, Ningbo NO.2 Hospital, NO.41 Xibei Street, Ningbo City, 315000 Zhejiang Province China

**Keywords:** Papillary thyroid cancer, Recurrence, Biomarker, miR-221

## Abstract

**Background:**

Many studies have reported several transcriptionally deregulated microRNAs (miRNAs) in papillary thyroid cancer (PTC) tissue in comparison with benign thyroid nodules and normal thyroid tissues. However, the correlation between miRNA expressions and PTC recurrence still remains unclear.

**Methods:**

The PTC patients who scheduled to undergo total thyroidectomy by the same surgical team in Ningbo NO.2 Hospital from March 1998 to March 2008 were enrolled in this study. The clinical and pathological characteristics of each patient were recorded in detail. The selected miRNA expressions were detected using quantitative reverse transcriptase-polymerase chain reaction (qRT-PCR). Potential predictive factors for cancer recurrence were evaluated by univariate and multivariate Cox proportional hazard analysis.

**Results:**

A total of 78 patients were enrolled with 49 females at a mean age of 45.8 years. Enrolled patients were divided into two groups: nonrecurrent group (*n* = 54) and recurrent group (*n* = 24). The results from the univariate Cox proportional hazard analysis revealed that primary tumor size, TNM stage, extrathyroid extension, miR-221, and miR-222 expressions were significantly associated with PTC recurrence (*P* < 0.05). The tissue expression of miR-221 was the only independent risk factor for PTC recurrence (HR 1.41; 95%CI 1.14–1.95, *P* = 0.007) by multiple Cox proportional hazard analysis.

**Conclusions:**

This study identified the potential role of miR-221 as a prognostic biomarker for the recurrence in PTC.

## Background

Thyroid cancer is the most prevalent and rapidly increasing endocrine neoplasm. The number of new cases is almost 300,000 worldwide with a median age at diagnosis of 50 years and nearly 40,000 deaths per year [1]. The most common histological type of all thyroid cancers is papillary thyroid cancer (PTC), which is defined as a differentiated neoplasia and accounts for approximately 80%. Most PTC patients are with good prognosis, and the 10-year survival rate is about 90% [2]. However, the incidence of lymph node metastasis can be as high as 20–50% [3], and PTC patients undergoing total thyroidectomy are with a regional recurrence of 5–20% [4, 5]. Currently, needle aspiration biopsy is the gold standard for diagnosis, but the predicative value for recurrence is rather limited [6]. Risk factors for the prognosis and recurrence of PTC are controversial among different current guidelines [7, 8]. Therefore, investigating potential useful markers for the identification and distinction of recurrence risk factors is extremely imperative and necessary [9].

MicroRNAs (miRNAs), small noncoding single-stranded RNAs, exert important actions in the development and metastasis of cancer as reported by previous studies [10, 11]. Some miRNAs have already been suggested as potential prognostic markers for the evaluation of cancer types, stages, or progression [12, 13]. Recent studies have also observed several transcriptionally deregulated miRNAs in PTC tissue in comparison with benign thyroid nodules and normal thyroid tissues [14, 15]. However, the correlation between miRNA expressions and PTC recurrence still remains unclear.

Previous studies have frequently reported that some miRNAs including miR-21, miR-9, miR-10b, miR-146b, miR-31, miR-220, miR-221, and miR-222 are abnormally expressed in thyroid cancers compared with healthy controls [16–19]. In this present study, we investigate the potential role of these miRNAs in the recurrence of PTC.

## Methods

### Patients

This study protocol was approved by the Medical Institutional Ethics Committee of Zhejiang province. The PTC patients who scheduled to undergo total thyroidectomy by the same surgical team in Ningbo NO.2 Hospital from March 1998 to March 2008 were eligible to enter this study. All patients included were required to offer written informed consent. The formalin-fixed, paraffin-embedded (FFPE) PTC tissues were procured for the postoperative histopathologic diagnosis and miRNA measurements. Those patients with poor quality of tissues, missed follow-up data, or with no signed informed consent were excluded from this study. A total of 78 patients were enrolled with 49 females (62.8%) at a mean age of 45.8 years. Enrolled patients were divided into two groups: nonrecurrent group (*n* = 54) and recurrent group (*n* = 24). Only those patients with no recurrence after a 120-month follow-up evaluated by clinical, laboratorial, and radiological evidence were categorized in nonrecurrent group. Recurrence in this present study was defined as locoregional recurrence or distant metastasis. The median follow-up period was 68 (range 8–158) months.

### Methods

Selective neck dissections were performed in PTC patients with regional lymph node metastases considering the metastases location. Our practice including the radioactive iodine (RAI) and surgical management evolved according to the latest American Thyroid Association (ATA) guidelines. All the patients were followed up through outpatient department clinic regularly over time. The conduction of postoperative RAI therapy was under the direction of the ATA guidelines [7]. Two independent pathologists blinded to this study were required for the evaluation of tumor histopathologic features. The ATA classification, risk stratification by Memorial Sloan Kettering Cancer Center (MSKCC–NY) [20], and stage by Union for International Cancer Control (UICC) TNM [21] were also evaluated. The clinical and pathological characteristics of each patient were recorded in detail.

### Tissue samples and RNA extraction

In this study, we use the areas containing over >90% malignant tissue annotated by the same experienced pathologist as the available PTC tissue samples. Manual macrodissection was performed in archival FFPE blocks for RNA extraction. Approximately 5–10-um-thick sections from the FFPE PTC tissues were obtained for total RNA extraction by utilizing RNeasy Kit (Qiagen, Hilden, Germany) according to the manufacturer’s instructions. NanoDrop ND1000 Spectrophotometer (ThermoFisher Scientific, Waltham, Mass) was used for the assessment of RNA quality and concentration.

### qRT-PCR for miRNA measurement

The miRNA expressions were detected using quantitative reverse transcriptase-polymerase chain reaction (qRT-PCR) with TaqMan miRNA assays (Applied Biosystems, Foster City, Calif) and the △△Ct method. In brief, TaqMan miRNA primers were used for the synthesis of cDNA from total RNA, following the manufacturer’s instructions. The TaqMan miRNA probes and a 7500 Real-time PCR System were utilized for amplification of PCR products. Two PCR reactions were performed per sample following the manufacturer’s protocol. RNU48 expression levels were used as an endogenous control for data normalization [22]. As for those cases of multifocal PTCs, the sample obtained from available multifocal PTC tissues was all subjected to miRNA qRT-PCR. The final data were described with the mean value.

### Statistical analysis

SPSS 21.0 (SPSS, Inc.) was utilized for data analysis in this study. Data were presented as number (*n*) and percentage (%), or mean ± standard error (SE) when appropriate. We used chi-square or Fisher’s exact tests to analyze clinical categorical data as appropriate. Continuous data was analyzed by Student’s *t* tests or Mann-Whitney *U* tests. Potential predictive factors for cancer recurrence were evaluated by univariate and multivariate Cox proportional hazard analysis, and statistical difference was set as *P* < 0.05.

## Results

### Patient characteristics and PTC recurrence

A total of 78 eligible patients with a mean age of 45.8 years and mean tumor size of 24.5 (range 8–47) mm were enrolled in this study with signed informed consent. Fifty-four of all the included patients were categorized into nonrecurrent group with a mean age of 44.9 years. 

It has been observed that those patients with higher ATA risks, higher MSKCC–NY risks, a larger primary tumor size, or higher TNM stages were more likely to result in the PTC recurrence (*P* < 0.05) (see Table 1). Moreover, the patients in recurrent group were also with a higher percentage of extrathyroid extension and cervical lymph node metastasis (*P* < 0.05).

**Table 1:** Characteristics of patients and PTC recurrence

Parameters	Recurrent group (*n* = 24)	Nonrecurrent group (*n* = 54)	*P* value
Age (year)	44.9 ± 22.5	46.2 ± 18.9	0.792
Sex			
Male	5(%)	13(%)	0.754
Female	19(%)	41(%)	
ASA physical status			
I	8(%)	20(%)	0.673
II	11(%)	27(%)	
III	5(%)	7(%)	
BMI (kg/m^2^)	21.4 ± 4.3	20.7 ± 3.8	0.473
ATA risk			
Low	6	28	0.030*
Intermediate	14	24	
High	4	2	
MSKCC–NY risk			
Low	4	11	0.017*
Intermediate	10	36	
High	10	7	
Primary tumor size (mm)	29.4 ± 5.4	22.3 ± 4.8	<0.001*
Lymph node dissection	15(62.5%)	37(68.5%)	0.603
RAI postoperatively	6(25.0%)	22(40.7%)	0.181
Recurrence place			
Locoregional	22(%)	47(%)	
Distant metastasis	2(%)	7(%)	0.713
Serum thyroglobulin (ng/mL)	1.6 ± 1.4	1.0 ± 1.3	0.071
TNM stage			
I–II	13	43	0.021*
III–IV	11	11	
Extrathyroid extension			
Yes	15	17	0.010*
No	9	37	
Vascular invasion			
Yes	11	18	0.292
No	13	36	
Perineural invasion			
Yes	6	7	0.188
No	18	47	
Cervical lymph node metastasis			
Yes	7	6	0.048*
No	17	48	
Histological subtype			
Classic	19	42	0.891
Follicular	5	12	
Multifocal tumors			
Yes	13	19	0.116
No	11	35	
Bilateral tumors			
Yes	7	16	0.967
No	17	38	

*P* values were calculated by chi-square test, Fisher’s exact test, Student’s *t* test, or Mann-Whitney *U* tests

*PTC* papillary thyroid cancer, *ASA* American Society of Anesthesiologists, *BMI* body mass index, *ATA* American Thyroid Association, *MSKCC–NY* Memorial Sloan Kettering Cancer Center

*P value <0.05

### Tissue miRNA expressions and PTC recurrence

To investigate the potential associations between tissue miRNA expressions and PTC recurrence, we evaluated miR-21, miR-9, miR-10b, miR-146b, miR-31, miR-220, miR-221, and miR-222 by qRT-PCR. As shown in Table 2, miR-146b, miR-220, miR-221, and miR-222 were markedly higher, while miR-9 and miR-21 expressions were significantly lower in recurrent group when compared with the nonrecurrent group (*P* < 0.05).

**Table 2:** Univariate and multiple Cox proportional hazard analysis between clinical and pathological variables with PTC recurrence

	Univariate		Multivariate	
Variables	HR(95%CI)	*P* value	HR(95%CI)	*P* value
ATA risk				
Low × high	1.67(0.38–7.64)	0.389		
Low × intermediate	0.87(0.29–3.35)	0.879		
Intermediate × high	3.34(1.42–6.75)	0.087		
MSKCC–NY risk				
Low × high	1.67(0.77–3.98)	0.196		
Low × intermediate	2.12(0.78–5.86)	0.132		
Intermediate × high	1.45(0.52–3.88)	0.243		
Primary tumor size	5.14(3.14–11.64)	0.009*	1.54(0.43–7.67)	0.567
TNM stage				
I/II × III/IV	4.31(2.01–9.87)	0.011*	2.68(1.10–6.72)	0.067
Extrathyroid extension	2.12(0.78–6.81)	0.021*	2.53(0.54–12.42)	0.314
Cervical lymph node metastasis	2.85(0.76–6.12)	0.107		
miR-21	2.01(0.33–5.43)	0.132		
miR-9	1.32(0.54–4.53)	0.225		
miR-146b	1.11(0.24–4.13)	0.682		
miR-220	1.13(0.45–4.14)	0.745		
miR-221	1.46(1.20–1.88)	0.001*	1.41(1.14–1.95)	0.007*
miR-222	2.81(1.11–7.21)	0.021*	1.86(0.76–5.65)	0.226

*PTC*, papillary thyroid cancer, *ATA* American Thyroid Association, *MSKCC–NY* Memorial Sloan Kettering Cancer Center, *CI* confidence interval, *HR* hazard ratio

*P value <0.05

### Univariate and multiple Cox proportional hazard analysis for PTC recurrence

**Fig. 1:**
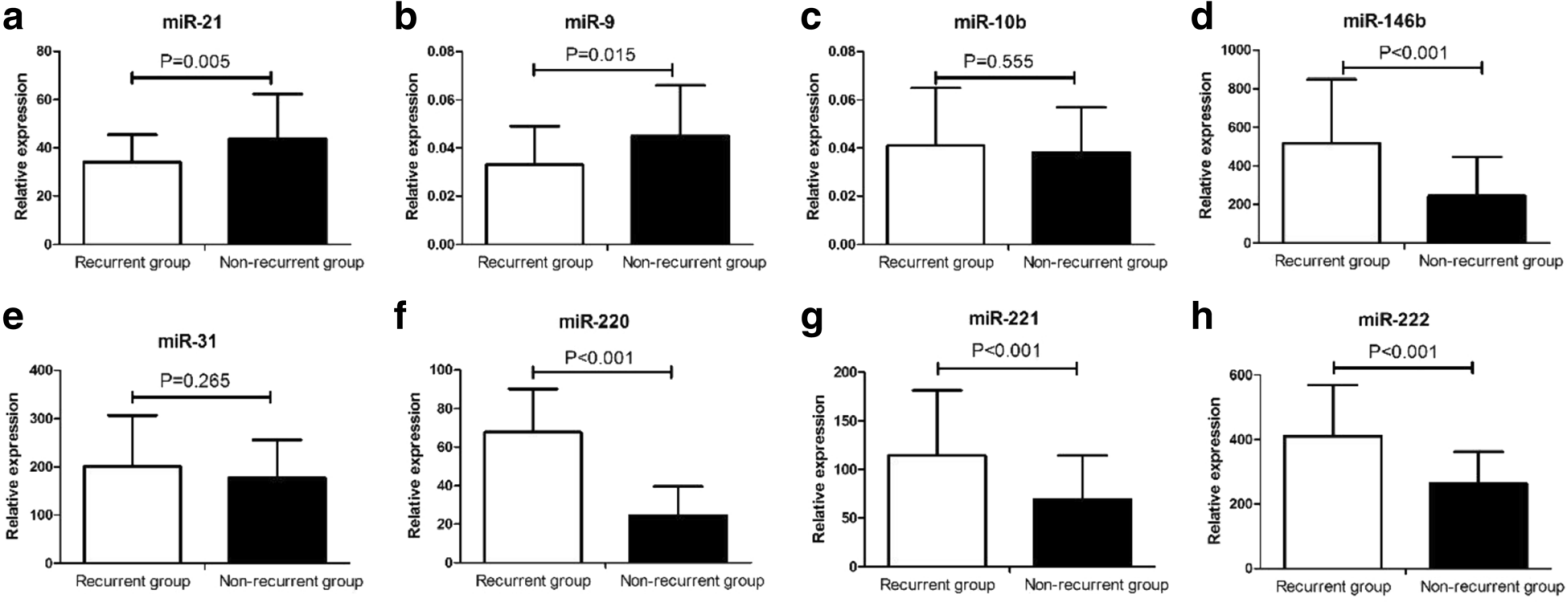
Relative expressions of tissue microRNAs i﻿ncluding miR-21 (**a**), miR-9 (**b**), miR-10b (**c**), miR-146b (**d**)﻿, miR-31 (**e**), miR-220 (**f**), miR-221 (**g**) and miR-222 (**h**) in papillary thyroid cancer (PTC) patients with or without recurrence

The univariate and multiple Cox proportional hazard analysis was utilized for the investigation of potential variables for predicating PTC recurrence. The all potential recurrence-associated variables including clinical, pathological parameters, and tissue miRNA expressions (Table 1 and Fig. 1) were involved into Cox proportional hazard model. The results from univariate Cox proportional hazard analysis revealed that primary tumor size, TNM stage, extrathyroid extension, miR-221, and miR-222 expressions were significantly associated PTC recurrence (*P* < 0.05). The tissue expression of miR-221 was the only independent risk factor for PTC recurrence (HR 1.41; 95%CI 1.14–1.95, *P* = 0.007) by multiple Cox proportional hazard analysis (Table 3).

**Table 3:** Relative miR-221 expressions and recurrence-associated parameters

	Relative expressions of miR-221		
Parameters	High (*n* = 12)	Low (*n* = 12)	*P* value
Disease-free interval (year)	1.8 ± 4.7	1.3 ± 5.3	0.801
Thyroglobulin (ng/mL)	1.5 ± 1.0	0.6 ± 0.9	0.031*
Cervical lymph node metastasis	5/12	2/12	0.371

*P* values were calculated by Mann-Whitney *U* tests or Fisher’s exact test

*P value <0.05

### MiR-221 and other recurrence-associated parameters

No close association between miR-221 levels and time to recurrence was found by linear analysis. As shown in Table 3, the patients with higher miR-221 expressions (>median level) were associated with higher thyroglobulin levels. There was no significant association between miR-221 levels with disease-free interval and cervical lymph node metastasis rate.

## Discussion

The valid predicative biomarkers for PTC recurrence would help adequate assessment of PTC patients, less prophylactic lymph node resections, or extensive surgeries. Whether prophylactic cervical lymph node dissection could improve postoperative survival or prevent PTC recurrence still remains controversial [23, 24]. However, no such effective biomarkers have been found until now, and great efforts are carried out by researchers for this purpose. This present study aimed at investigating potential biomarkers for PTC recurrence including tissue miRNA expressions.

Previous studies have revealed that larger tumor size was associated with increased incidence of nodal spread and worse prognosis [25, 26]. Several reports have also suggested tumor size as a predictor for central lymph node metastases in PTC patients [27]. However, our final multiple Cox analysis did not support these factors as predictive factors for PTC recurrence.

Previous studies have revealed the oncogenic and tumor suppressor roles of miRNAs during the formation and progression of tumors through the pathway modulation. Furthermore, several miRNAs have been reported closely correlated with the proliferation, progression, metastasis, and invasion of tumors [12]. Some researchers also suggest some miRNAs may exert predictive roles in cancer [13]. However, whether tissue or serum miRNAs could actually assist in the predication of PTC recurrence is still unclear. In this study, we examined a panel of tissue miRNA expressions to investigate potential biomarkers for PTC recurrence by qRT-PCR. The tissue expressions of miR-9, miR-21, miR-146b, miR-220, miR-221, and miR-222 in recurrent group were significantly different from the nonrecurrent group. The downregulated miR-9 and miR-21 expressions in recurrent group comparing with nonrecurrent group were in accordance with other studies [1]. MiR-146b is also reported overexpressed in PTC tissues and closely associated with high-risk features including BRAF mutation or extrathyroidal invasion [28]. Overexpressed miR-221 and miR-222 in PTC are closely correlated with clinic-pathological characteristics and tumor aggression [15, 19]. As reported by previous studies, miR-220 was deregulated in the PTC tissues vs. the normal tissues [14]. Our analysis demonstrated that miR-220 expressions were significantly higher in recurrent group compared with nonrecurrent group. In this present study, we indicated miR-221 expressions in PTC samples as potential biomarkers for PTC recurrence. Excessively secreted miR-221 levels have been observed in PTC, follicular, and anaplastic thyroid cancers [29]. Previous studies also indicate that excessive expressions of miR-221 act as an important role in the proliferation of thyroid cancer cells [14]. Sehu S et al. also report the significantly different expressions of miR-221 between benign follicular adenoma, multinodular goiter, and PTC samples [30]. All these findings strongly suggest a close correlation between miR-221 and PTC.

Some findings have revealed the crucial role of miR-221 in the occurrence or progression of human osteosarcoma and suggested miR-221 as a promising marker for the diagnostic and prognostic potentials for osteosarcoma [31]. MiR-221 was also recommended as a significant prognostic factor of clear cell renal cell carcinoma [32]. As one of the most sensitive miRNAs for PTC [33], miR-221 was identified as a predictive biomarker for PTC recurrence in this study. Enhanced expression of miR-221 and miR-222 plays a critical role in melanoma progression by activating fundamental pathways, such as blocking melanogenesis and inducing cell survival [34]. F. Acibucu et al. indicate that upregulated miR-221 can promote breast cancer progression by suppressing E-cadherin expression [35]. MiR-221 is established as a tumor suppressor for prostate cancer uniquely by targeting Runx2 [36]. These may be possible molecular mechanisms of that miR-221 lead to increased cancer growth and progression.

## Conclusions

In conclusion, this study identified miR-221 as a significant independent predictor for PTC recurrence. However, our study also had some limitations. First, a long-term follow-up in the current study and larger cohorts are required to support miR-221 as a useful biomarker for PTC recurrence in clinical practice. Second, the ATA guidelines changed throughout the collection period, and our practice including the RAI and surgical management evolved according to the latest ATA guidelines. Whether the changed RAI and surgical management would affect our results remained unclear. Third, the involved mechanisms why miR-221 expression could serve as potential predicative factor for recurrence in PTC still remained unknown.

## Abbreviations

ASA: American Society of Anesthesiologists
ATA: American Thyroid Association
BMI: Body mass index
CI: Confidence interval
FFPE: Formalin-fixed, paraffin-embedded
HR: Hazard ratio
MSKCC–NY: Memorial Sloan Kettering Cancer Center
PTC: Papillary thyroid cancer
qRT-PCR: Quantitative reverse transcriptase-polymerase chain reaction
RAI: Radioactive iodine
